# Association of *OPRD1* Gene Variants with Changes in Body Weight and Psychometric Indicators in Patients with Eating Disorders

**DOI:** 10.3390/jcm13175189

**Published:** 2024-09-01

**Authors:** Laura González-Rodríguez, Luz María González, Angustias García-Herráiz, Sonia Mota-Zamorano, Isalud Flores, Guillermo Gervasini

**Affiliations:** 1 Department of Medical & Surgical Therapeutics, Medical School, University of Extremadura, 06006 Badajoz, Spain; lgonzrodrig@gmail.com (L.G.-R.); lmgonzalezgarcia89@gmail.com (L.M.G.); smotazamorano@gmail.com (S.M.-Z.); 2 Eating Disorders Unit, Health Service of Extremadura, 06010 Badajoz, Spain; angusgaher1@gmail.com (A.G.-H.);; 3 Institute of Molecular Pathology Biomarkers, University of Extremadura, 06010 Badajoz, Spain

**Keywords:** anorexia, bulimia, eating disorders, opioids, *OPRD1*

## Abstract

**Objectives**: This study aimed to investigate whether genetic variations in the *OPRD1* gene affect psychopathological symptoms and personality dimensions in eating disorders (ED) patients and/or contribute to ED risk. **Methods**: The study involved 221 female patients with anorexia nervosa (AN), 88 with bulimia nervosa (BN), and 396 controls. Sixteen tag-single nucleotide polymorphisms (SNPs) in *OPRD1* were identified. Psychometric evaluations were conducted using the Symptom Checklist 90 Revised (SCL-90R) and the Eating Disorders Inventory Test-2 (EDI-2). *p*-values obtained by regression models were corrected for multiple testing by the False Discovery Rate (FDR) method. **Results**: In AN patients, genotypes rs204077TT and rs169450TT were linked to lower body-mass index (BMI) values (FDR-q = 0.035 and 0.017, respectively), as was rs2234918 in a log-additive model (BMI: 18.0 ± 0.28, 17.22 ± 0.18 and 16.59 ± 0.39 for TT, TC and CC carriers, FDR-q = 0.012). Additionally, AN patients carrying the rs72665504AA genotype had higher scores in interpersonal distrust (FDR-q = 0.030), whilst BN carriers of rs513269TT and rs2873795TT showed lower scores in ineffectiveness (FDR-q = 0.041 and FDR-q = 0.021). In the AN group, BMI correlated with variability in a distal haplotype (rs508448/rs204077/rs223491, FDR-q = 0.028), which was also associated with the global positive symptom total (PST) index of SCL-90R (FDR-q = 0.048). Associations were more noticeable in BN patients; again, the distal region of the gene was linked to EDI-2 total scores (FDR-q = 0.004–0.048 for the four last haplotypes) and two global SCL-90R indices (GSI: FDR-q = 0.011 and positive symptom distress index (PSDI): FDR-q = 0.003 for the last s204077/rs2234918/rs169450 combination). No associations with ED risk were observed. **Conclusions**: Genetic variation in the *OPRD1* gene, particularly in its distal region, is associated with BMI and psychopathological comorbidities in ED patients.

## 1. Introduction

Eating disorders (EDs) are serious mental health conditions characterized by disturbed eating habits that can significantly impact physical health, psychological well-being, and overall quality of life. These disorders, anorexia (AN) and bulimia nervosa (BN) in particular, have become one of the most frequent chronic diseases among young females in the western hemisphere. The situation has worsened since the COVID-19 pandemic, with more than a 50% increase in the number of young women hospitalized because of EDs [1]. Genetics play an important role in EDs; indeed, large-scale twin studies have revealed high heritability estimates for both AN and BN, while genome-wide association studies (GWAS) have also identified relevant loci for these disorders [2]. Among the pathways of interest, the endogenous opioid system has been related to the onset and progression of ED [3]. These opioids play an important role in the regulation of energy balance and eating behavior, and dysregulation of this system results in maladaptive dietary responses in ED patients [4]. The intimate involvement of the opioid system, with reward mechanisms underlying addictions, may also explain the connection with ED, given the rewarding effects of food consumption. In this regard, positron emission tomography (PET) imaging studies have revealed an increased opioid tone in brain areas of AN patients [5], and opioid antagonists are being evaluated for the treatment of ED [6]. It has been proposed that malfunctions in the systems regulating opioid activity, such as those that would arise from the presence of variants in the genes involved, would make carriers more susceptible to developing a self-addiction to exercise, food restriction, or lead to an abnormal craving for the rewarding effects of binge eating [4].

Early linkage studies in AN patients and unaffected relatives identified a region of chromosome 1 that was significant for the disease containing, among others, the *OPRD1* gene coding for the delta opioid receptor [7]. Subsequently, three GWAS and follow-up studies identified several *OPRD1* variants that could be associated with AN status [8,9,10], although conflicting results also exist [11]. Furthermore, animal studies have shown that opioid peptides considered for the treatment of anorexia have orexigenic effects that are mediated by the delta receptor [12], and that delta receptor agonists influence feeding behavior [13]. On the other hand, the connection between genetics and ED may also be explained by the impact that genetic variants have on personality traits and psychopathological symptoms that are known predisposing factors of these disorders [14,15]. For instance, we have previously shown that variability in cannabinoid [16] and dopamine receptors [17,18], also involved in reward mechanisms, or in serotonin receptors [19] is associated with psychiatric comorbidities in ED patients.

This background, which highlights the importance of the endogenous opioid system in eating behavior and provides indications that the delta receptor in particular might play an important role in ED, prompted us to study whether genetic variation in *OPRD1*, estimated by 16 identified tag-single nucleotide polymorphisms (SNPs) capturing the variability of the whole gene locus, could contribute to the risk for ED and/or affect psychopathological symptoms and personality traits that are commonplace in these disorders.

## 2. Materials and Methods

### 2.1. Study Design

**Figure 1:**
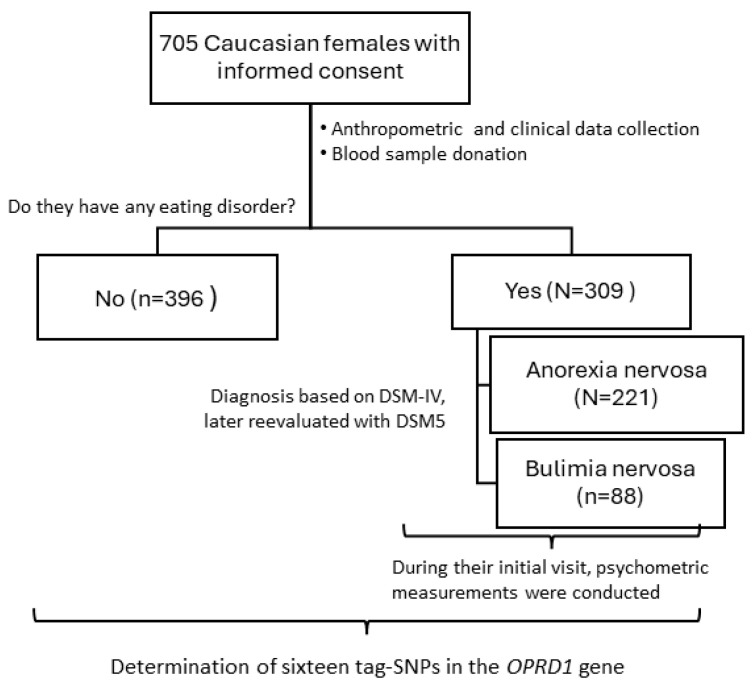
Study design. SNP, single nucleotide polymorphisms.

This study cohort comprised 221 caucasian female patients with anorexia nervosa (AN), without a prior history of bulimia nervosa (BN), and 88 patients with BN, all recruited from the Eating Disorders Unit of the Extremadura Health Service in Badajoz, Spain, over a period of five years (Figure 1). Additionally, DNA samples were also obtained from 396 female caucasian Spanish donors who had a normal body-mass index (BMI) and no history of psychiatric or endocrine disorders. These samples were stored at the Carlos III DNA National Bank at the University of Salamanca, Spain. All participants were diagnosed by a psychiatrist and a psychologist in their first visit using the Eating Disorders section of the Structured Clinical Interview for the Diagnostic and Statistical Manual of Mental Disorders (DSM)-IV, and diagnoses were later reassessed to align with DSM-5 criteria. All patients (or their legal guardians) provided written informed consent for study participation and were also interviewed to collect psychometric data (details below) in this first visit. Exclusion criteria included conditions such as schizophrenia, intellectual disability, dementia, Turner’s syndrome, or other endocrine or neurological diseases. The study received approval from the Bioethics Committee of the University of Extremadura and was conducted following the Declaration of Helsinki and its subsequent amendments.

### 2.2. Tag-SNPs Selection and Genotype Analysis

We extracted the coding and adjacent 3′ and 5′ untranslated regions (UTR) sequences of the *OPRD1* gene (ENSG00000116329; HGNC:8153) to identify tag-single nucleotide polymorphisms (tag-SNPs) using Haploview 4.2 software (Cambridge, MA, USA). Tag-SNPs are index genetic variants in regions with high linkage disequilibrium (LD), representing groups of variants inherited together. The selection criteria included a pair-wise tagging, with r^2^ > 0.80, and a minor allele frequency threshold of 10%. The sixteen tag-SNPs identified in this manner are listed in Table 1. Whole blood samples (10 mL) were collected from the patients and stored at −80 °C until DNA extraction, which was performed using a standard phenol-chloroform technique. Genotyping was conducted at the Centro Nacional de Genotipado (CeGen) in Santiago de Compostela, Spain, utilizing an OpenArray customized panel on a QuantStudio™ 12K Flex Real-Time PCR System (Life Technologies, Carlsbad, CA, USA).

### 2.3. Personality Dimensions and Psychopathological Symptoms

All patients completed two self-report questionnaires during their initial visit to the unit, namely the Symptom Checklist 90 Revised (SCL-90R) and the Eating Disorders Inventory Test-2 (EDI-2). The SCL-90R evaluates a wide array of psychopathological symptoms across nine categories (somatization, obsessive–compulsive, interpersonal sensitivity, depression, anxiety, hostility, phobic anxiety, paranoid ideation, and psychoticism) as well as three global indices: the Global Severity Index (GSI), the Positive Symptom Distress Index (PSDI), and the Positive Symptom Total (PST). The SCL-90R has been validated for use in Spain, demonstrating robust reliability for its scales [20]. The EDI-2 focuses on psychological and behavioral traits commonly associated with ED, covering eight primary scales: drive for thinness, bulimia, body dissatisfaction, ineffectiveness, perfectionism, interpersonal distrust, interoceptive awareness, and maturity fears. Additionally, Asceticism, Impulse Regulation, and Social Insecurity were later included. The EDI-2 questionnaire has also demonstrated high internal consistency in the Spanish population [21].

### 2.4. Statistical Analyses

Results from the psychometric evaluation with EDI-2 and SCL-90R are shown as mean ± standard deviation (SD) values. Student’s *t*-tests or Mann–Whitney tests, depending on data distribution assessed by the Kolmogorov–Smirnov method, were used to compare quantitative variables between groups. To identify genetic associations with clinical and anthropometric variables, logistic regression models adjusted for age were conducted using the SNPassoc R (v. 4.4.1) package. In order to identify broader areas within the *OPRD1* gene locus that could modulate BMI and/or the psychometric results of the ED patients, we used a sliding-window approach to construct successive and adjacent 3-SNP haplotypes, establishing a minimum frequency threshold of 0.1 using PLINK v1.07 software. Statistical power calculations were conducted by determining the frequency of each variant with a log-additive model and an assumed effect size of 2.0 (type-I error = 0.05). In the case of the risk analysis, the numbers of controls per case were 1.28, 1.79, and 4.5 for ED, AN, and BN, respectively. The statistical power ranged from 0.801 to 0.957, depending on the minor allele frequency and specific ED (Quanto software v. 1.2.4, University of Southern California, Los Angeles, CA, USA). The *p*-values obtained were adjusted for multiple comparisons by the Benjamini–Hochberg False Discovery Rate (FDR) method, considering the number of SNPs and haplotypes analyzed. An FDR-q value < 0.05 was considered statistically significant.

## 3. Results

Table 2 summarizes the clinical and descriptive characteristics of the participants in this study. As expected, AN patients showed lower weight, body mass index (BMI), and were younger than subjects in the BN group (*p*-value < 0.05 in all cases). In addition, BN patients scored significantly higher than AN patients in the different scales measured in the psychometric evaluation (*p*-value < 0.001). The minor allelic frequencies of the 16 tag-SNPs studied in the OPRD1 gene in the whole population of study varied from 0.203 to 0.469. None of these genetic variants showed significant deviations from the Hardy–Weinberg equilibrium (Table 1).

### 3.1. Single-Marker Regression Analyses

**Figure 2:**
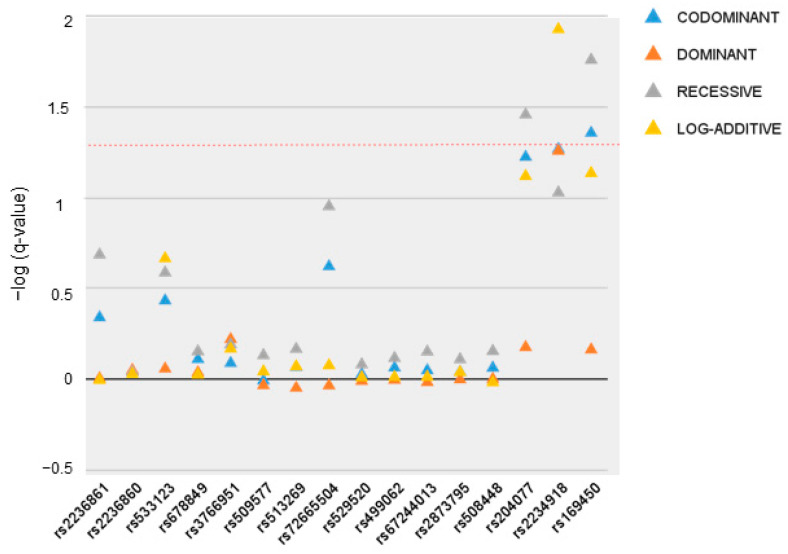
Significance levels of regression analyses in all models of inheritance for the 16 SNPs assayed in relation to body-mass index values of Anorexia Nervosa patients. The red dotted line represents the 0.05 adjusted q-level of significance.

First, we examined whether any of the tag-SNPs were related to significant differences in the BMI of ED patients. In the AN group, two genotypes, rs204077 TT and rs169450 TT, were associated with lower BMI values (16.00 ± 0.46 vs. 17.51 ± 0.15 vs. the other two genotypes, FDR-q = 0.035 and FDR-q = 0.017, respectively). Mean values are equal for the two variants because subjects with the TT homozygous variant genotype were the same in both cases. Rs2234918 was also associated with lower BMI in a log-additive model (18.0 ± 0.28, 17.22 ± 0.18, and 16.59 ± 0.39 for TT, TC, and CC, respectively, FDR-q = 0.012). Figure 2 shows the regression analysis in all models on inheritance for all 16 SNPs assayed in AN patients. In the BN group, none of the genotypes analyzed retained statistical significance after correction for multiple testing (Supplementary Materials Figure S1).

**Figure 3:**
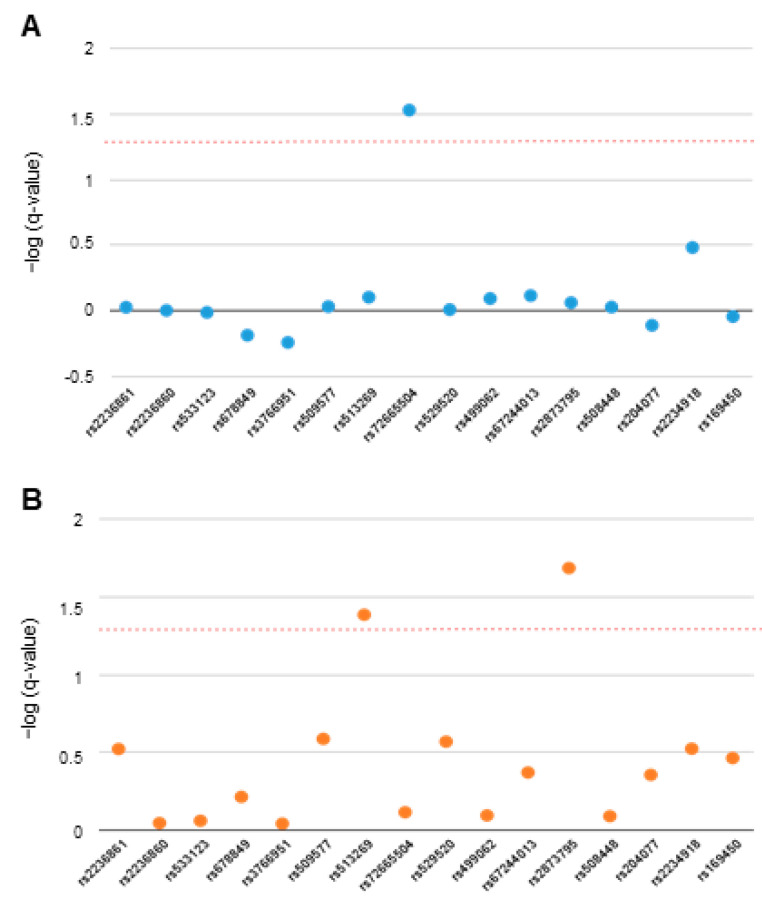
Impact of *OPRD1* tag-SNPs on interpersonal distrust scores in anorexia patients (**A**) and on ineffectiveness in bulimia patients (**B**) obtained with a recessive model of inheritance. The red dotted line represents the 0.05 adjusted q-level of significance.

Next, we evaluated the impact of variability in the OPRD1 gene on the psychometric scores obtained by ED patients. After correction for multiple comparisons, AN patients carrying the rs72665504 AA genotype scored significantly higher than GG/AG carriers on the interpersonal distrust dimension of the EDI-2 inventory (FDR-q = 0.030). In BN patients, carriers of the rs513269 TT and rs2873795 TT variant genotypes showed lower scores than the other two genotypes in the Ineffectiveness scale of the same questionnaire (FDR-q = 0.041 and FDR-q = 0.021, respectively). Figure 3 shows all FDR-corrected q-values for associations with these two scales of the EDI2 inventory in AN and BN patients, respectively.

With regard to the risk analysis, rs67244013 and rs72665504 were found to be associated with the development of AN. Nevertheless, none of these genotypes retained statistical significance after correction for multiple testing. None of the SNPs analyzed were found to be individually associated with BN risk. Supplementary Materials Tables S1 and S2 show all *p*-values obtained for the assayed SNPs. Key findings obtained in this single-marker analysis are summarized in Table 3.

### 3.2. Multiple-Marker Analysis—Sliding-Windows Approach

**Figure 4:**
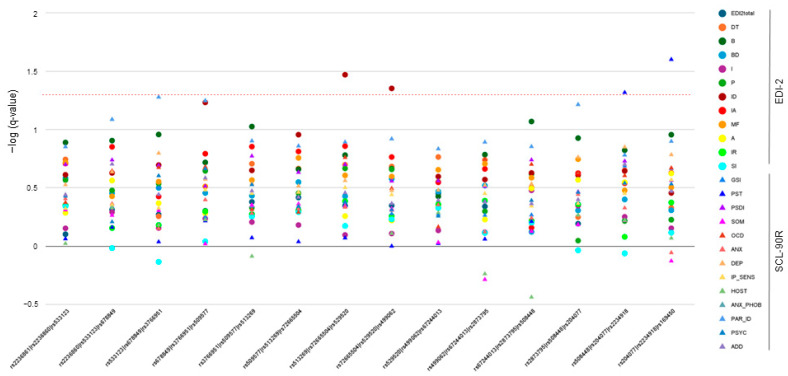
Three-SNP sliding-window analysis for the association of *OPRD1* haplotypes with psychometric scores of AN patients. The dotted line denotes the 0.05-adjusted q-level of significance. EDI2total, EDI-2 total score; DT, drive for thinness; B, bulimia; BD, body dissatisfaction; I, ineffectiveness; P, perfectionism; ID, interpersonal distrust; IA, interoceptive awareness; MF, maturity fears; A, asceticism; IR, impulse regulation; SI, social insecurity; GSI, Global Severity Index; PST, Positive Symptom Total; PSDI, Positive Symptom Distress Index; SOM, Somatization; OCD, obsessive–compulsive; ANX, anxiety; DEP, depression; IP_SENS, interpersonal sensitivity; HOST, hostility; ANX_PHOB, phobic anxiety; PAR_ID, paranoid ideation; PSYC, psychoticism; and ADD, additional items.

To further investigate these observations, we performed a sliding-window analysis to identify the associations of the resulting 14 3-SNP haplotypes with both BMI and psychometric scores. In the AN group, BMI correlated significantly with variability in a distal rs508448/rs204077/rs223491 haplotype (FDR-q = 0.028). Figure 4 displays the values for the 3-SNP combinations with psychometric scores in AN patients. As it occurred in the single marker study, the interpersonal distrust dimension of the EDI-2 inventory showed significant associations with rs513269/rs72665504/rs529520 (FDR-q = 0.034) and rs72665504/rs529520/rs499062 haplotypes (FDR-q = 0.044). In addition, the global PST index of the SCL-90R test was also associated with the two last 3-SNP combinations, namely rs508448/rs204077/rs2234918 (FDR-q = 0.048) and rs204077/rs2234918/rs169450 haplotype (FDR-q = 0.025).

**Figure 5:**
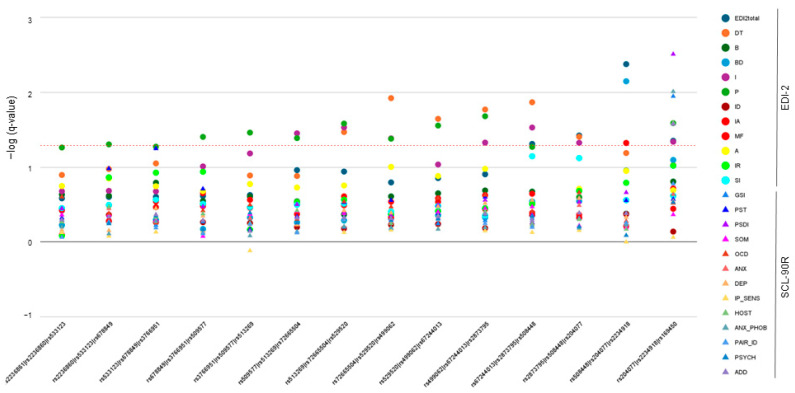
Three-SNP sliding-window analysis for the association of *OPRD1* haplotypes with psychometric scores of BN patients. The dotted line denotes the 0.05 adjusted q-level of significance. EDI2total, EDI-2 total score; DT, drive for thinness; B, bulimia; BD, body dissatisfaction; I, ineffectiveness; P, perfectionism; ID, interpersonal distrust; IA, interoceptive awareness; MF, maturity fears; A, asceticism; IR, impulse regulation; SI, social insecurity; GSI, Global Severity Index; PST, Positive Symptom Total; PSDI, Positive Symptom Distress Index; SOM, Somatization; OCD, Obsessive–Compulsive; ANX, anxiety; DEP, depression; IP_SENS, interpersonal Sensitivity; HOST, hostility; ANX_PHOB, phobic anxiety; PAR_ID, paranoid ideation; PSYC, psychoticism; and ADD, additional items.

With regard to the BN group, whereas there were no significant results for BMI after FDR correction, the degree of the associations with the psychometric scores gradually increased towards the distal part of the gene (Figure 4). Most importantly, this area was strongly linked to the total EDI-2 score (FDR-q values ranging from 0.004 to 0.048 for the four last haplotypes) and two general indices of SCL-90R (FDR-q = 0.011 for GSI and FDR-q = 0.003 for PSDI for the last s204077/rs2234918/rs169450 combination). In general, EDI-2 measurements were much more affected by the variability in *OPRD1*. In particular, ineffectiveness (as observed in the single-marker analysis), Perfectionism, and drive for thinness were three dimensions showing significant associations throughout the entire gene locus. The lowest FDR-q-value obtained was 0.007 for the association between Body dissatisfaction and the rs508448/rs204077/rs2234918 haplotype (Figure 5).

## 4. Discussion

A great challenge in the study on the role of genetics in ED is that, like in most psychiatric disorders, its influence seems to follow a non-Mendelian patter, i.e., there is a great number of genes in various regions of the genome that may play a significant role [22]. *OPRD1*, coding for the delta opioid receptor, has been suggested to be one such gene [23], as the receptor signaling is believed to contribute to the activity of dopaminergic neurons in the ventral tegmental area, affecting reward mechanisms in the mesolimbic circuit, thus regulating hunger, satiety, and hedonic eating [4]. However, to date, there are no genetic association studies assessing its influence on anthropometric or psychometric characteristics of ED patients.

Our findings show that a distal region of the *OPRD1* gene locus, encompassing tag-SNPs rs204077, rs2234918, and rs169450, was significantly associated with lower BMI values in AN patients. Previous studies on *OPRD1* genetic variability have focused on AN risk, but none has assessed the impact on BMI. Interestingly, Paszynska et al. have shown that the levels of opiorphin, a peptide implicated in the rapid inactivation of endogenous opioids correlate with body weight in AN patients [24], thus suggesting that changes in opioid levels may translate into BMI changes, most likely because of an alteration in the reward system that regulates food intake. Our findings also support this hypothesis, indicating that genetic variability in *OPRD1* could affect the delta receptor function and hence affect reward stimuli. However, we used a tag-SNP design in this study, meaning that the precise effects of the variants might be due to other non-synonymous SNPs in high LD. In any case, we were able to pinpoint an area in *OPRD1* within chromosome 1 (positions 1:28862203 to 1:28871216) that seems to be important for BMI in AN patients.

The analysis of ED-related comorbidities as potential intermediate phenotypes, may help determine the biological mechanisms involved in ED and identify genetic variabilities contributing to these disorders [25]. Bodnar et al. have recently reviewed the important role of endogenous opioids in modulating behavior [26]. In this regard, both our single- and multiple-marker approaches identified a number of traits and psychopathological symptoms in AN and BN patients that were affected by variability in the *OPRD1* gene. To our knowledge, there are no targeted studies that analyze the role of opioid genes in these ED-related comorbidities, although there are several reports revealing associations of *OPRD1* variants with addictive behavior, a characteristic that can be seen in some ED patients [27]. Specifically, these studies link *OPRD1* variability to the response to pharmacological treatment for opioid dependence and the risk of addiction to opioids and cocaine [28,29]. There is only a GWAS that analyzed the Twins UK cohort dataset for associations with phenotypes related to ED [30]. The authors reported that *OPRD1* rs1042114 was associated with body dissatisfaction. Interestingly, we also observed a strong association (FDR-q = 0.007) between this trait and a 3-SNP haplotype formed by rs508448, rs204077, and rs2234918 in patients with BN, a disorder in which body image disturbances constitute a core feature [31]. Although information on the biochemical effects of tag-SNPs is typically scarce, the first SNP in the haplotype, rs508448, is in LD with rs1042114, a non-synonymous coding variant that has been shown to affect the delta opioid receptor function [32]. In addition, the third variant in the haplotype, rs2234918, has been well studied, and there are reports linking its presence to the modulation of oxycodone effects [33] and heroin dependence [34]. It should be noticed that these two tag-SNPs with putative functional impact on the receptor activity are contained in the last four consecutive 3-SNP haplotypes identified. This is important because looking at Figure 3 and Figure 4, it seems clear that most of the significant associations with the psychometric measurements arise from variability in this distal region of the gene locus, where the aforementioned variants are harbored. Remarkably, the results from the BN group showed that this area was associated with altered scores in the total EDI-2 questionnaire as well as in two global indices of the SCL-90R inventory, GSI and PSDI, which highlights the importance of this distal region in the *OPRD1* gene for the development of psychiatric comorbidities that are often coupled with ED. Interestingly, in the AN group, the associations with PST, the third general distress index of SCL-90R, and with BMI were also located in this region. It is true, however, that, in general, less noticeable genotype–phenotype associations were identified in the AN patients. An explanation could be that differences in the questionnaires’ results due to an expected moderate influence of genetic variants would be more noticeable in patients with elevated scores. In this regard, BN patients were found to score significantly higher than AN patients in all scales, as patients with BN have a higher prevalence of comorbidity with other psychopathological disorders [35]. Furthermore, connections among genotypes of genes in the central nervous system and these traits have been shown to be stronger in BN patients than in AN patients [36].

The findings described herein do not support that *OPRD1* genetic variability is a key element in the risk for AN or BN, as only two SNPs in the AN group showed statistical significance, which was lost after correction for multiple testing. In this regard, it should be noted that a GWAS conducted to identify genetic modulators of AN risk did not find SNPs with genome-wide significance [10]. The authors did report an association for rs533123 in *OPRD1*, which we did not observe, but it was only nominal; hence, the evidence must be considered suggestive. Brown et al. have also reported a significant association with AN risk for rs569356, an SNP that was not included in our tag-SNP design; although, it should be noted that the authors did not correct their results for multiple testing [9]. In previous studies of our group, we have observed how the connection of central nervous genes with ED susceptibility might be considered unconvincing, whereas there is a much clearer association with personality dimensions and psychopathological symptoms shown by the patients, which can translate, for instance, into BMI changes [36,37,38,39]. It is tempting to speculate that the altered traits are likely more important with respect to the severity and specific characteristics of the ED than to an elevated susceptibility, where sociocultural or environmental elements may also play a relevant role.

Naltrexone, an opioid antagonist, is FDA-approved for opiate and alcohol use disorders as an anti-craving agent, but it has also been used successfully off-label in other psychiatric indications [40]. Its use in ED has also been proposed and is being evaluated in ongoing clinical trials including ED patients with or without binge-eating [41]. Our results, which argue for a key role of *OPRD1* in traits related to ED, provide additional evidence supporting this therapeutic strategy, as modulation of the delta opioid receptor could help the management of these disorders through the improvement of associated psychiatric comorbidities, which are frequently as protracted and impairing as the ED itself [42].

A few limitations must be acknowledged in the present work. In the first place, the limited size of the BN group might affect the reproducibility of its associated findings. On the other hand, the limited sample allowed that all the patients were caucasians living in the same geographical area and that were diagnosed and treated by the same doctors in a single healthcare center, all of which reduced the chance that the findings may be due to population structure. Second, we did not include the numerous personality dimensions assessed in the correction for multiple testing, as we did with the genetic variants and allele combinations determined. Finally, connecting the observed associations to a precise biochemical effect of a given SNP is challenging, as the study design included the use of tag-SNPs, which often lack functional information.

## 5. Conclusions

In summary, we conclude that genetic variability in the *OPRD1* gene, and specifically in the distal region of the gene locus (from position 1:28862203 onwards), plays a significant role in modulating BMI, personality dimensions, and psychopathological symptoms in patients with ED. These findings suggest that alterations in the reward system regulated by the delta opioid receptor may contribute to the regulation of body weight and the severity of psychiatric comorbidities in ED. Even though our study did not identify a central role of *OPRD1* in the overall risk of developing AN or BN, the identified genetic associations with BMI and psychiatric symptoms, which were more noticeable in BN patients, highlight its importance in the nuanced expression of ED phenotypes and underscore the need for further research to elucidate the precise biochemical mechanisms underlying these associations.

## Figures and Tables

**Table 1:** Tag-SNPs analyzed in the study.

Tag-SNP	Alleles	Position	MAF	HWE
rs2236861	G/A	1:28813244	0.394	0.586
rs2236860	C/T	1:28814236	0.355	0.819
rs533123	A/G	1:28814643	0.408	0.758
rs678849	C/T	1:28818676	0.25	0.483
rs3766951	T/C	1:28843047	0.433	0.531
rs509577	A/C	1:28845884	0.458	0.545
rs513269	C/T	1:28846339	0.394	0.208
rs72665504	G/A	1:28847410	0.465	0.530
rs529520	A/C	1:28848434	0.203	0.420
rs499062	T/C	1:28848540	0.219	1.000
rs67244013	G/A	1:28848988	0.469	0.168
rs2873795	G/T	1:28850273	0.289	0.208
rs508448	A/G	1:28855013	0.356	0.474
rs204077	C/T	1:28862203	0.448	0.329
rs2234918	T/C	1:28863085	0.334	0.543
rs169450	G/T	1:28871216	0.255	0.828

HWE, Hardy–Weinberg equilibrium in the control population; MAF, minor allele frequency in the study population.

**Table 2:** Descriptive and psychometric features of patients with anorexia nervosa or bulimia nervosa and healthy females. Mean ± standard deviation values are shown.

Characteristics	Anorexia Nervosa	Bulimia Nervosa	Eating Disorders	Controls
N	221	88	309	396
Age, years	17.0 ± 4.1	18.7 ± 5.9 ^ʃ^	17.5 ± 4.7 *	32.6 ± 8.0
Weight, kg	45.0 ± 7.0	68.1 ± 22.3 **	51.5 ± 16.8 *	63.1 ± 7.7
BMI, kg/m^2^	17.3 ± 2.1	25.9 ± 8.2 **	19.7 ± 6.1 *	23.4 ± 2.72
Height, m	1.61 ± 0.07	1.62 ± 0.06	1.61 ± 0.07 *	1.64 ± 0.06
Total EDI-2	89.9 ± 46.3	121.4 ± 41.0 **	98.8 ± 47.0	-
GSI	1.6 ± 0.8	2.0 ± 0.8 **	1.7 ± 0.8	-
PST	61.1 ± 21.5	70.4 ± 16.76 **	63.8 ± 20.6	-
PSDI	2.2 ± 0.6	2.4 ± 0.6 **	± 0.6	-

* p-value < 0.05 vs. control subjects; ^ʃ^ *p*-value < 0.05 vs. anorexia patients; ** *p*-value < 0.001 vs. anorexia patients. BMI, body-mass index; EDI-2, Eating Disorders Inventory Test-2; Global Symptom Index; PST, Positive Symptom Total; PSDI, Positive Symptom Distress Index.

**Table 3:** Main findings obtained in the single-marker analysis.

Group/Feature	Associated Variation	FDR-q
Anorexia Nervosa		
Lower BMI	rs204077 TT	0.035
Lower BMI	rs169450 TT	0.017
Lower BMI	rs2234918-C allele	0.012
Interpersonal distrust	rs72665504 AA	0.030
Risk	None	
Bulimia Nervosa		
Ineffectiveness	rs513269 TT	0.041
Ineffectiveness	rs2873795 TT	0.021
Risk	None	

BMI, body-mass index.

## Data Availability

The data that support the findings of this study are available on Figshare with DOI accession number https://www.doi.org/10.6084/m9.figshare.26303329 (accessed on 15 August 2024).

## Supplementary Materials

The following supporting information can be downloaded at: https://www.mdpi.com/article/10.3390/jcm13175189/s1. Figure S1: Significance levels of regression analyses in all models of inheritance for the 16 SNPs assayed in relation to body-mass index values of Bulimia Nervosa patients. Table S1: *p*-values for the association of 16 tag-SNPs in *OPRD1* with the risk of developing Anorexia Nervosa. All models of inheritance are shown. Table S2: *p*-values for the association of 16 tag-SNPs in *OPRD1* with the risk of developing Bulimia Nervosa. All models of inheritance are shown.
