# Supplementary material for: Association of OPRD1 Gene Variants with Changes in Body Weight and Psychometric Indicators in Patients with Eating Disorders
Source: J Clin Med. 2024 Sep 1;13(17):5189. doi: 10.3390/jcm13175189 (PMC11396092; doi:10.3390/jcm13175189)
Supplement: Supplementary file 1 [file jcm-13-05189-s001.zip › SUPPLEMENTARY TABLES.pdf]

**SUPPLEMENTARY TABLES**

**Table S1.** P-values for the association of 16 tag-SNPs in *OPRD1* with the risk of developing Anorexia Nervosa. All models of inheritance are shown.

| tag-SNPs | MAF controls | MAF patients | codominant | dominant | recessive | log-additive |
|---|---|---|---|---|---|---|
| rs2236861 | 0.242 | 0.278 | 0.579 | 0.303 | 0.883 | 0.379 |
| rs2236860 | 0.323 | 0.372 | 0.530 | 0.497 | 0.517 | 0.863 |
| rs533123 | 0.203 | 0.187 | 0.764 | 0.996 | 0.475 | 0.833 |
| rs678849 | 0.484 | 0.441 | 0.485 | 0.229 | 0.740 | 0.328 |
| rs3766951 | 0.399 | 0.441 | 0.346 | 0.879 | 0.193 | 0.520 |
| rs509577 | 0.471 | 0.432 | 0.214 | 0.103 | 0.907 | 0.306 |
| rs513269 | 0.398 | 0.385 | 0.758 | 0.739 | 0.460 | 0.545 |
| rs72665504 | 0.274 | 0.332 | 0.076 | 0.023 | 0.515 | 0.045 |
| rs529520 | 0.466 | 0.464 | 0.583 | 0.300 | 0.675 | 0.357 |
| rs499062 | 0.255 | 0.231 | 0.412 | 0.306 | 0.277 | 0.207 |
| rs67244013 | 0.206 | 0.256 | 0.122 | 0.040 | 0.622 | 0.062 |
| rs2873795 | 0.398 | 0.385 | 0.904 | 0.702 | 0.729 | 0.654 |
| rs508448 | 0.436 | 0.439 | 0.607 | 0.440 | 0.388 | 0.322 |
| rs204077 | 0.361 | 0.335 | 0.521 | 0.533 | 0.269 | 0.324 |
| rs2234918 | 0.455 | 0.434 | 0.643 | 0.720 | 0.464 | 0.873 |
| rs169450 | 0.362 | 0.335 | 0.525 | 0.529 | 0.274 | 0.324 |

MAF, minor allele frequency

**Table S2.** P-values for the association of 16 tag-SNPs in OPRD1 with the risk of developing Bulimia Nervosa. All models of inheritance are shown.

| tag-SNPs | MAF controls | MAF patients | codominant | dominant | recessive | log-additive |
|---|---|---|---|---|---|---|
| rs2236861 | 0.242 | 0.256 | 0,681 | 0,470 | 0,779 | 0,626 |
| rs2236860 | 0.323 | 0.29 | 0,383 | 0,193 | 0,418 | 0,168 |
| rs533123 | 0.203 | 0.239 | 0,343 | 0,433 | 0,164 | 0,252 |
| rs678849 | 0.484 | 0.472 | 0,552 | 0,316 | 0,936 | 0,555 |
| rs3766951 | 0.399 | 0.364 | 0,305 | 0,309 | 0,143 | 0,145 |
| rs509577 | 0.471 | 0.466 | 0,602 | 0,315 | 0,818 | 0,430 |
| rs513269 | 0.398 | 0.398 | 0,876 | 0,627 | 0,996 | 0,732 |
| rs72665504 | 0.274 | 0.25 | 0,599 | 0,493 | 0,621 | 0,738 |
| rs529520 | 0.466 | 0.46 | 0,852 | 0,572 | 0,843 | 0,634 |
| rs499062 | 0.255 | 0.278 | 0,772 | 0,868 | 0,474 | 0,665 |
| rs67244013 | 0.206 | 0.188 | 0,312 | 0,530 | 0,224 | 0,848 |
| rs2873795 | 0.398 | 0.403 | 0,824 | 0,557 | 0,996 | 0,679 |
| rs508448 | 0.436 | 0.403 | 0,298 | 0,646 | 0,204 | 0,710 |
| rs204077 | 0.361 | 0.386 | 0,563 | 0,290 | 0,853 | 0,394 |
| rs2234918 | 0.455 | 0.455 | 0,712 | 0,495 | 0,834 | 0,747 |
| rs169450 | 0.362 | 0.369 | 0,471 | 0,241 | 0,989 | 0,390 |

MAF, minor allele frequency
