# Supplementary figures and images for: Association of OPRD1 Gene Variants with Changes in Body Weight and Psychometric Indicators in Patients with Eating Disorders
Source: J Clin Med. 2024 Sep 1;13(17):5189. doi: 10.3390/jcm13175189 (PMC11396092; doi:10.3390/jcm13175189)

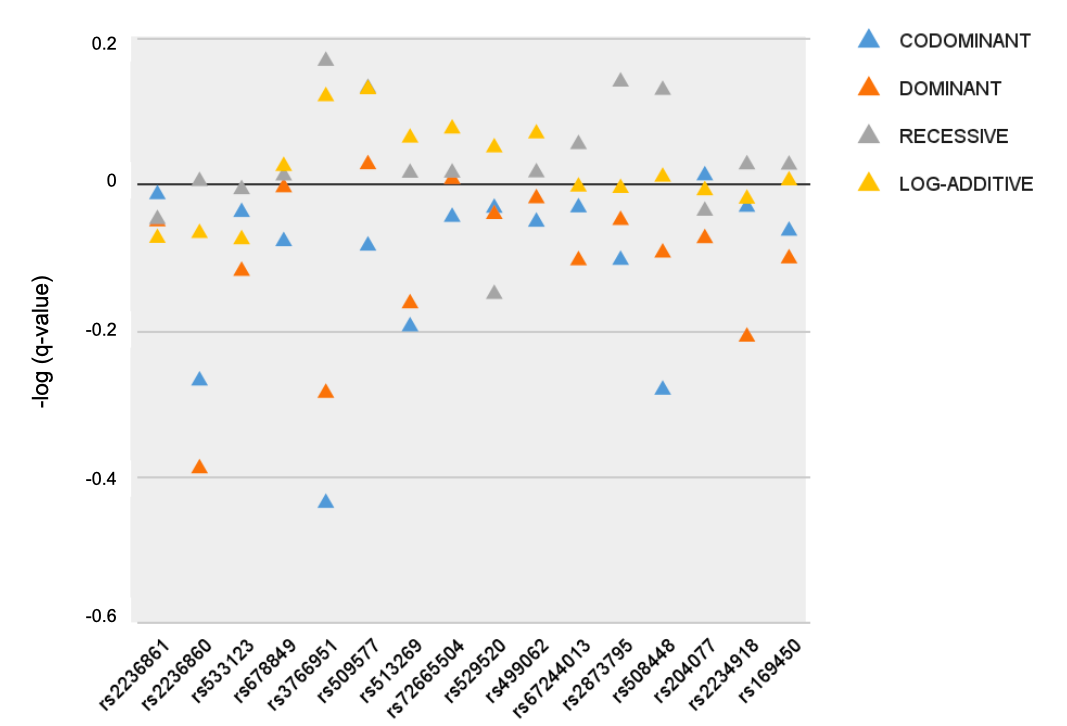

Supplement: Supplementary file 1 [file jcm-13-05189-s001.zip › Figure S1.tif]
